# New Cork-Based Materials and Applications

**DOI:** 10.3390/ma8020625

**Published:** 2015-02-10

**Authors:** Luís Gil

**Affiliations:** Laboratório Nacional de Energia e Geologia, I. P., Estrada do Paço do Lumiar, 22, Edf. C, 1649-038 Lisboa, Portugal; E-Mail: luis.gil@lneg.pt; Tel.: +351-210-924-757

**Keywords:** cork, composites, cork applications, mechanical properties, material properties

## Abstract

This review work is an update of a previous work reporting the new cork based materials and new applications of cork based materials. Cork is a material which has been used for multiple applications. The most known uses of cork are in stoppers (natural and agglomerated cork) for alcoholic beverages, classic floor covering with composite cork tiles (made by the binding of cork particles with different binders), and thermal/acoustic/vibration insulation with expanded corkboard in buildings and some other industrial fields. Many recent developments have been made leading to new cork based materials. Most of these newly developed cork materials are not yet on the market, but they represent new possibilities for engineers, architects, designers and other professionals which must be known and considered, potentially leading to their industrialization. This paper is a review covering the last five years of innovative cork materials and applications also mentioning previous work not reported before.

## 1. Introduction

Cork is the bark of the cork oak tree (*Quercus suber* L.). This suberous material is composed of layers of small cells, more than 40 million per cubic centimeter. These cells have five layer walls made of cellulose, lignin, suberin, tannins, waxes. Cork is a cellular natural material, very versatile, very light in weight, elastic, flexible, impermeable to gases or liquids, and good electric insulator, as well as a thermal, sound and vibration insulator and also a dielectric material. Its unique properties arise from its closed cell structure [1,2].

Southern Mediterranean countries are the world’s major producers of cork, mainly Portugal, which holds about 1/3 of the total cork tree area and produces about 50% of cork at world level. The last data refer about 2,100,000 ha of cork oak forests and a cork production of about 201,000 t/year [3]. Cork oak forests are extremely well adapted to Europe’s southern and Africa’s northern semi-arid regions. These forests prevent desertification, give rise to other environmental benefits (CO_2_ sequestration, hydrological cycle, *etc.*) and are an important habitat for many animal and plant species.

Cork physical-mechanical characteristics make it an excellent material for thermal insulation, most advantageously, e.g., in cold chambers where compressive loads are present, and also for acoustic absorption (e.g., recording studios) and vibration insulation (e.g., machinery). Its pleasant sensation to the touch, energy absorbing and anti-sliding properties make it also good for coverings, shoes or in handles. Its compression-recovery properties make it the material of choice for seals and joints in civil construction, woodwind instruments and combustion engines and, of course, as stoppers [1,2].

New and innovative cork materials must be known and considered by engineers, architects, designers and other professionals. Cork composites are one of the most promising fields of cork technology evolution. A review of progress in the last years, selecting some of the most representative of these materials and new applications, most of which are not yet in the market and are interesting for multiple purposes, follows.

## 2. New Cork Materials and Applications

### 2.1. Cork Based Sandwich and Plywood Materials

Cork based agglomerates are being considered an interesting core material for sandwich components, mainly of lightweight, high-performance and low-maintenance structures with specific properties. Some consider that current core materials have low structural freedom and, in certain cases, high environmental burdens. Therefore, it has been suggested that benefits in performance and economic and environmental aspects could be achieved by hybrid sandwich panels comprising non-traditional materials as cork based materials [4,5]. Cork has properties which allow a better performance regarding damage tolerance due to impact loads [6]. For this, low speed impact tests were performed and residual strength characterization through four-point bending tests. Damage extension quantification was carried out showing evident advantages relatively to other types of core materials.

A cork-based plastic composite material has been proposed [4] and its mechanical properties, economic benefit and environmental impact in comparison with glass fiber reinforced plastic investigated. The application in light, stiff panels was also investigated. The results proved that the cork composite is competitive with other core materials, e.g., wood-based or other plastic core materials.

Carbon fiber-synthetic foam core sandwich composites typically have very poor acoustic performance and there is an increasing demand for noise mitigation. Therefore, a study was carried out, showing the association of carbon fiber composites with natural cork in a sandwich structure. This association provides a synergistic effect yielding a sandwich composite with good noise behavior without sacrificing mechanical performance and weight, while also achieving a 250% improvement in damping performance with increased durability and lifetime operation [7]. The transition from synthetic foam to cork also aims at environmental friendly materials and a reduction in carbon footprint. Improvements are foreseen in acoustic and vibrational performance in applications such as aircraft cabins or wind turbine blades.

In order to analyze the viability of using cork-based materials as core materials in sandwich structures in aeronautical and aerospace applications, a study on the mechanical behavior of different materials was carried out [8]. The cork-based materials were proposed because of their isolation properties (thermal, acoustic) with no significant performance loss in comparison to current materials. Cork is proposed also due to less energy manufacturing processes and better environmental integration (transformation, recycling). Experimental shear tests and three-point bending tests were used in the study, showing that some improvements can be made. A previous work [9] also looked at the viability of applying cork-based materials in aeronautical and aerospace applications as core materials in sandwich structures.

In another study [5] two different types of cork layered plywood composites, a plywood board with a cork core and a plywood with a cork core and cork face layers, were produced and tested (mechanical properties). The measured properties were compared with those of standard particleboard and standard plywood. The results showed that the cork layered plywood had superior mechanical properties at a much lower density than the particleboard. In comparison to plywood, reductions in density and production costs were observed.

A patented cork core for being placed between two surface skins of a sandwich panel was developed [10]. The core has at least two overlapped cork agglomerate layers defining regular macrocavities. Another core material [11] for a sandwich panel comprising a composite combining a thermoplastic resin and cork powder was also developed. The product was made by injection-molding.

### 2.2. Cork Based Damping Materials

Cork agglomerates and cork rubber composites available in the market and with a wide range of compositions may prove to be the solution for a material providing damping capacity but with the additional characteristics of low mass density and thermal and acoustic insulation. A new study [12] on their elastic-dynamic properties using dynamic mechanical analysis was carried out. The dynamic longitudinal modulus of some cork composite materials was measured, showing the mechanical response of these materials to a sinusoidal stress or strain in the linear viscoelastic regime. The materials behavior depends on the frequency and temperature. These two parameters influence the dynamic modulus and this is sensitive to the composition and structure of the composites, so they can be useful for correlations with the performance and for design purposes.

Composition cork can also be an interesting solution for light-damped sandwich panels. Selected composition cork materials were comparatively studied for vibration damping applications [13]. The results show an air spring/viscous based mechanism ruling the low frequency behavior of these materials.

### 2.3. Absorbent Cork Based Materials

A study [14] on the possibility of using cork materials (macroporous solids with very low density) as biosorbents and precursors of activated carbons, aiming at achieving added value to byproducts of cork processing was carried out. The cork material was submitted to impregnation with phosphoric acid followed by pyrolysis under nitrogen. The biosorbents were treated with a cationic surfactant and activated carbons were submitted to another pyrolysis under propene, which enhanced the affinity for oil in the pores. A physical-chemical, textural and surface characterization of the materials was then carried out. Samples were selected for testing in the removal of oil emulsified in water. The conclusion was that re-granulated cork sorbents and phosphoric acid activated carbons can be applied for the removal of emulsified oil from water.

A review on the use of cork powder and granules for the adsorption of pollutants (gaseous emission, waters and wastewaters, heavy metals, oils, VOCs (volatile organic compounds)) can be found in [15].

A new product was launched onto the market with the trade name of CorkSorb to help control the oil spills [16]. The oil is captured by capillarity and kept inside the cork cells. Cork is hydrophobic, absorbing oils and solvents but not water. This product (cork granules) is mentioned as having a much greater absorption capacity (9.43 L/kg) compared to mineral absorbents. The producer refers that its market potential is very high.

### 2.4. Cork-Plastic Composites

There is a growing trend in the use of lignocellulosic materials as filler and/or reinforcement agent in plastic composites. Cork-polymer composites (CPC) are a promising field related to sustainable development and this was the subject of a Ph.D. thesis [17]. When cork is combined with a polymer matrix, new fields of application arise. High density polyethylene and polypropylene were combined with cork through melt based technologies to produce cork-based composites. Cork was combined with biodegradable aliphatic polyesters. Pultrusion and twin-screw extrusion and compression and injection molding processes were used for CPC production. Different reinforcement strategies were followed leading to cork based composites with better mechanical properties and improved interfacial cork-polymer adhesion. The findings in this thesis show that the CPC materials reveal the required: (i) dimension stability with reduced water absorption, (ii) homogeneous distribution and dispersion of the cork particles in the polymer matrix, (iii) improved fire resistance to the matrix, good thermal and acoustic insulation properties and (iv) an interesting range of mechanical properties. Cork also offers economic and environmental advantages over traditional inorganic reinforcements and fillers. Therefore, the combination of cork with polymeric matrices results in a significant added value to cork-based materials, with high potential for a wide range of innovative applications including building, transports, aeronautics, naval construction and furniture. This same author described [18] composites of cork powder (50% *w*/*w*) mixed with polypropylene or polyethylene, their physical-mechanical characterization and comparison with commercially available products such as medium and high density fiberboard.

A new composite produced by the company Greenfiber Tech [19] can also be included in this category. This is a kind of WPC (Wood Plastic Composites), but two wood fibers and cork are used (natural fiber composites) mixed with polypropylene, improving some technical characteristics. The applications are for outdoor furniture, decks and naval construction among others.

### 2.5. Densified Insulation Corkboard

**Figure 1:**
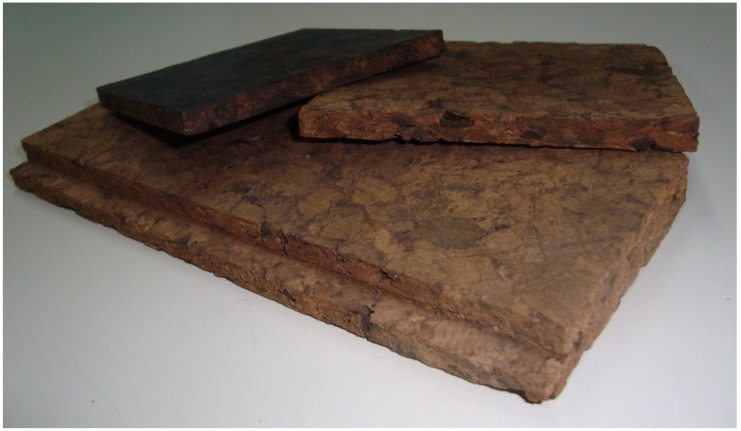
Samples of different densified insulation corkboard (ICB) materials.

This material (see Figure 1) is based on the densification of current insulation corkboard (ICB) also called expanded corkboard, which is a completely natural product, with no added binding agents. It is possible to produce this denser material with a wide range of operational conditions and therefore of products, with densities ranging from the maximum value possible for current ICB, usually from 250–300 kg/m^3^ to 750 kg/m^3^ or more. The densification of ICB is performed by heating the boards and hot pressing under pressure, temperature and time conditions such that irreversible densification is achieved. This has a smoother surface and better characteristics for new applications. Its manufacturing process is easy to adapt to current production and allows a diversification in production and in applications. Economic studies have shown that it can be competitive with some products on the market (e.g., wood-based materials, other cork-based materials). Some of the possible uses include floor coverings, wall and ceiling coverings, false (suspended) ceilings, screen and door panels, skirting boards, sandwich panels and furniture [20].

### 2.6. New Cork Applications in Transport Vehicles

**Figure 2:**
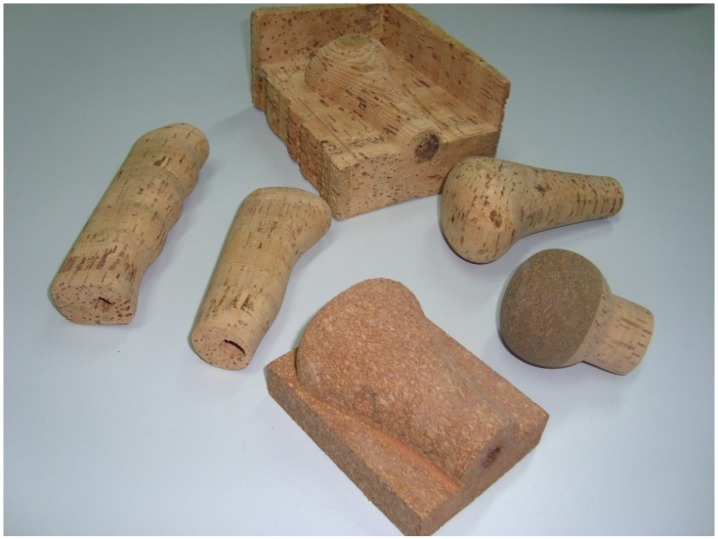
Gear and brake knobs made of cork.

**Figure 3:**
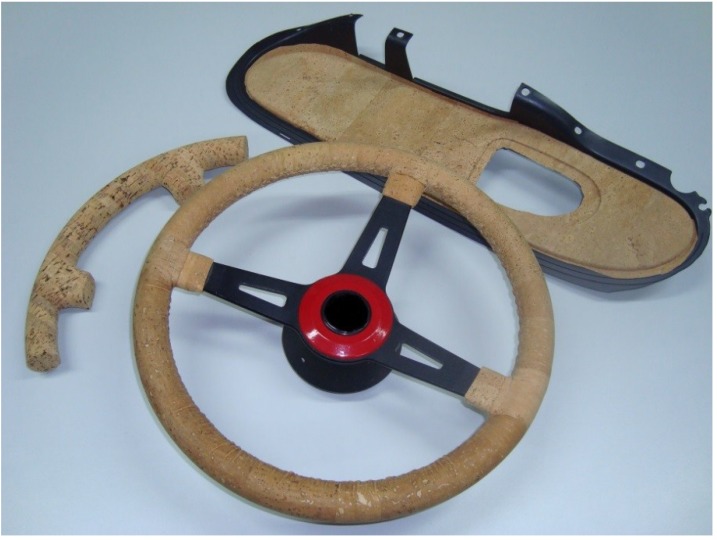
Steering wheel and door panel covered with corkskin.

The purpose of this work was to contribute to the recognition of the value of cork, through the utilization of this natural material in applications that will give rise to a greater visibility. This visibility brings about a higher added value in order to guarantee the sustainability of cork production and processing. The solutions proposed for this where new applications of cork in the automobile industry, namely in car interiors (Figure 2 and Figure 3). Based on natural cork or its derivatives, several prototypes of interior parts were made from solid cork or corkskin (leather type cork material). For example, the steering wheel and the gear knob produced with cork material present advantages in terms of thermal behavior and hence in increased comfort. Additionally, other solutions such as interior panels (e.g., door panels) and decorative elements that can contribute to the distinctive aesthetic characteristic to the vehicles’ interior were also considered. Cork materials used were corkskin, rubbercork and laminated cork blocks. Different technologies such as injection-molding, revolution abrasion, covering, CAD/CAM (computer-aided design and computer-aided manufacturing) were used, and operational conditions were optimized. Cork materials do not greatly vary their surface temperature when they are in extreme hot or cold environments and they are very agreeable to the touch. Costs of these materials are estimated to be very competitive to natural wood car parts. Tests were made measuring the surface temperature in gear knobs made of different materials, after being exposed at different temperatures for a certain period. Conclusions for negative and high temperatures (simulating a car interior in winter and in summer) are comparatively very good for cork [21,22].

The growing attention given to these factors (thermal behavior, decorative/luxury aspects) will induce a favorable reception to these solutions. Some automobile prototypes (e.g., Mercedes) have already been shown in car shows [23], but they were not mass produced.

New cars based on fuel cells, batteries or other engine based types, have usually a flat-bottom body, which is a good characteristic for cork floor applications in this field decreasing weight and carbon footprint.

New solutions for trains, trams and similar transportation vehicles, CoreCork^®^ and AluCork^®^ (sandwich panel made of CoreCork^®^, veneer and two thin aluminum layers), were developed for innovative interior systems (floor, lateral panels, ceiling panels). These solutions are based on the light weight and thermal and acoustic insulation properties of cork. The first product is a natural energy absorber for high impact strikes (rail front ends, impact prone areas on automobiles and trains and carriages). In canoes and kayaks, this has the ability to absorb rock and beach impact while also having an excellent compression recovery. It does not absorb water and does not rot and is resistant to fungal growth [24,25].

A project (Mould-cork) [26] is being carried out for the development of car parts (e.g., engine cover part) using cork composites in traditional car industry processes as stamping, injection and thermo-forming. It is predicted to decrease weight, and to increase thermal and acoustic insulation and also vibration damping, for better car performances, better comfort and emissions reduction.

### 2.7. Cork Microparticles as Reinforcement and Filler Agent

Structural adhesives have usually high strength and stiffness and low ductility and toughness. There are several processes to increase the toughness. A study was carried out [27] in which natural cork microparticles were used in order to increase the mechanical properties of a brittle epoxy adhesive. These particles, ranging from 125 to 250 μm and mixed with a chosen epoxy adhesive, act to prevent the propagation of cracks. Using tensile and impact tests it was evident that the mechanical properties were related to the ratio of cork particles:resin, considering an uniform particle distribution.

Cork industry produces a fine residue, cork powder, a light and granular waste material which should be valorized. Research [28] was carried out to look at the possibility of using this waste as filler in paper application. This brown cork granulate must firstly be refined to correct particle size distribution. Cork granulate can be incorporated at a maximum of 15% in weight in order to do not have a mechanical deleterious effect on inter-fiber links. The main advantage of using cork is ink control when using ink-jet printing inks, due to its porosity. It is possible to be applied in eucalyptus and pine fibers. One drawback is sheet color and it cannot be applied in paper with high brightness standards such as writing paper, but it can be used, e.g., in packaging paper and several other applications.

### 2.8. Design Products Made with Cork

**Figure 4:**
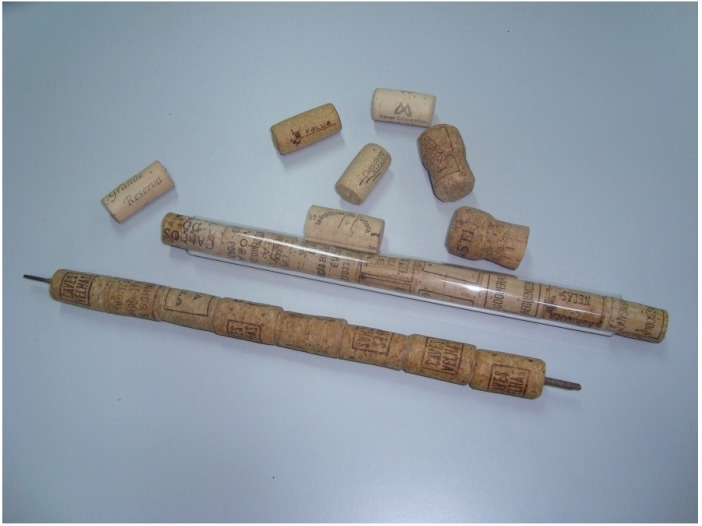
Batons of used cork stoppers.

A patented process was developed for the recycling of used cork stoppers and the production of utilitarian wares based on the gluing and transformation of these waste pieces [29]. Wine is drunk all over the world and its consumption is spreading to new markets. Cork processing plants are concentrated in the Atlantic-Mediterranean region, and even in these countries the most important places of consumption of bottled wine may be far away from the cork plants. In the countries where there is no cork industry, the volume of recovered used cork stoppers may be not enough to justify the installation of a new cork plant. Transportation of used stoppers from a long distance may be not ecologically viable. Hence, there was a need to develop a method for making good use of cork stoppers, while decreasing Municipal Solid Waste volume and treatment problems, at the place or near the place where the bottled wine is consumed. The process for the utilization of used cork stoppers is based on the regularization of the top and bottom of the stoppers, selection by diameter and gluing of corks top to bottom by means of a shaft or a tube (working as guides) in order to obtain a baton (Figure 4). The baton can be abraded by several means giving rise to utilitarian wares such as handles of lids and other kitchen wares, knobs and a lot of other products designers can imagine. These products can justify and promote the recovery of these waste products as they are easy to accept in the market and easy to produce. The process can be adapted by cork industry companies, artisans (craft) industries, cutlery industries and others [29,30].

Sustainable product design is a strong trend. In this context, cork, with its outstanding properties, can play a relevant role. Its versatility allows it to adopt different technological transformation processes and be used in different applications. An overview based on these aspects with a discussion centered on the gap between the research and implementation of cork materials and their application in new products is carried out in [31]. This work concludes that cork offers industrial designers a number of product-technology options to be used. Several possibilities of design products based on cork may be found in [32].

### 2.9. Cement Composites with Cork

Some recent investigations/commercial applications of cement composites with cork should also be mentioned, although this application is not in fact new. For example, a recent commercially available product, a light screed with incorporation of cork called ecoCORK [33] can be referred. This is a light mortar with incorporation of cork for the execution of filling and leveling layers on internal floors, with improved thermal and acoustic performance [34].

A study [35] examined the impact of cork used as sand or stone replacement on several properties of mortar and concrete. The influence of cork particles size, cork moisture saturation and cork percentage was studied. Another study [36] was carried out on the cyclic behavior of a lightweight mortar with cork granulate composite.

Lightweight cement-based screeds containing cork granulate waste were also developed [37]. The reduction of transmitted impact noise was assessed in two situations, using different cement dosages and different thicknesses. The results obtained show the potential of these composites in applications for reducing impact noise. The same authors carried out an experimental study [38] on the use of expanded cork granulate waste with cement-based mixtures in the production of screeds. These screeds were compared with mixtures without cork. Several properties were determined. Results show that the use of expanded cork granules decreases density, compressive strength and thermal conductivity of the screeds while increasing their water vapor permeability. The thermal delay of the concrete floors with layers of cork was analyzed [39]. The potential of these composites in applications for increasing the thermal performance was shown. A greater number of layers give rise to a higher thermal delay.

### 2.10. Other Specific Applications of Cork

**Figure 5:**
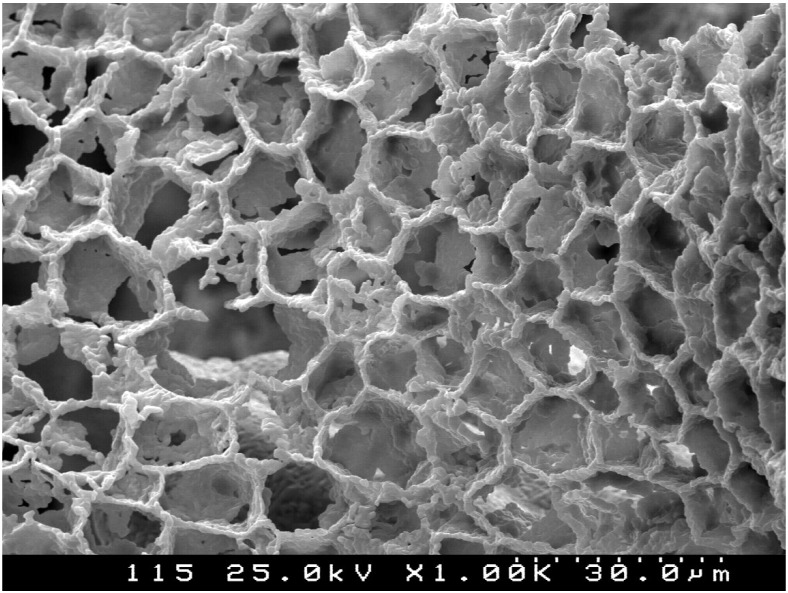
Magnetic hexaferrite ecoceramics with cork cellular microstructure (scanning electron microscope (SEM)) image courtesy of Pullar [31]).

For the first time ecoceramics (environmentally conscious ceramics) were produced based on cork, in this case using hexaferrites in order to obtain magnetic ceramic foams having the cork cellular structure. In this study [40] the ecoceramics were obtained by pyrolysis, using cork as a matrix and template, resulting in a ceramic material with the cork microstructure, solid, but very light and porous (Figure 5). Some interesting applications are predicted.

The potential use of waste cork materials for the production of activated carbons for adsorption of gases and liquids was reviewed [41]. It is possible to produce some carbon adsorbents with interesting structure and surface chemistry characteristics, comparable to those of commercial carbons. Several gases and volatile organic compounds were adsorbed and separated and the removal of phenolic and pharmaceutical compounds is possible. Production of composites and monoliths seems an alternative path. A recent work [42] shows that industrial pre-treated cork (granules of expanded cork) can be used as a precursor for the preparation of eco-friendly activated carbons by chemical and physical activation. These lab-made carbons have comparable properties to those obtained with samples for water treatment.

Cork granulate is now being used in artificial grass lawn for outdoor sports [43]. The cork granulate substitutes rubber in the filling of artificial lawns. The advantages of using cork are that it does not heat as much as rubber and is softer and it does not absorb water, causing fewer injuries in athletes and different ball bounce.

The use of cork composites in the internal covering of helmets was also studied [44]. The idea was to improve safety. Compared with expanded polystyrene, the usual material for this purpose, cork has a greater impact absorption capacity even under multiple impacts.

CORKwall^®^, a recent commercially available product [45], is a final coating for interior walls, ceilings, facades and roofs. This product is a cork-based emulsion applied through projection which acts as thermal and acoustic insulation. It can be used to repair and prevent cracks in the walls.

A patented synthetic clay composition [46] was also provided. It uses a viscosity controlling agent, cork powder and a binder resin.

## 3. Conclusions

A review and the description of new cork materials and applications were carried out. Several R&D results on cork derivatives are now waiting for the next step of industrialization. Cork derivatives are one of the most promising fields for cork technology development. Some possible applications are foreseen by the authors of the several referenced works. However, engineers, architects, designers and other professionals can and should also think in new possibilities, potentially leading to the production and use of these products.
